# DNA-intercalators Causing Rapid Re-expression of Methylated and Silenced Genes in Cancer Cells

**DOI:** 10.18632/oncotarget.863

**Published:** 2013-02-26

**Authors:** M. Zulfiquer Hossain, Megan A. Healey, Calvin Lee, Weijie Poh, Sashidhar R. Yerram, Kalpesh Patel, Nilofer S. Azad, James G. Herman, Scott E. Kern

**Affiliations:** ^1^ Sidney Kimmel Comprehensive Cancer Center, Department of Oncology, Johns Hopkins University School of Medicine, Baltimore, Maryland

**Keywords:** cancer, gene methylation, demethylation, DNA-intercalator, quinacrine, DNMT inhibitor, epigenetics, silencing and reactivation of gene expression, small molecule-DNA interactions

## Abstract

Epigenetic inactivation of tumor-suppressor and other regulatory genes plays a critical role in carcinogenesis. Transcriptional silencing is often maintained by DNA methyl transferase (DNMT)-mediated hypermethylation of CpG islands in promoter DNA. Nucleoside analogs including azacytidine and decitabine have been used to inhibit DNMT and re-activate genes, and are clinically used. Their shortcomings include a short half-life and a slow onset of action due to required nucleotide incorporation during DNA replication, which may limit clinical utility. It might be useful to begin to identify lead compounds having novel properties, specifically distinct and fast-acting gene desilencing. We previously identified chemicals augmenting gene expression in multiple reporter systems. We now report that a subset of these compounds that includes quinacrine re-expresses epigenetically silenced genes implicated in carcinogenesis. p16, TFPI2, the cadherins E-cadherin and CDH13, and the secreted frizzle-related proteins (SFRPs) SFRP1 and SFRP5 were desilenced in cancer cell lines. These lead compounds were fast-acting: re-expression occurred by 12-24 hours. Reactivation of silenced genes was accompanied by depletion of DNMT1 at the promoters of activated genes and demethylation of DNA. A model compound, 5175328, induced changes more rapidly than decitabine. These gene desilencing agents belonged to a class of acridine compounds, intercalated into DNA, and inhibited DNMT1 activity in vitro. Although to define the mechanism would be outside the scope of this initial report, this class may re-activate silenced genes in part by intercalating into DNA and subsequently inhibiting full DNMT1 activity. Rapid mechanisms for chemical desilencing of methylated genes therefore exist.

## INTRODUCTION

Genes silenced in cancer comprise tumor-suppressor genes, regulatory genes, and genes involved in differentiation. These genes are often inactivated by epigenetic mechanisms involving methylation of cytosines in CpG islands of promoter DNA, higher-order heritable chromatin folding/remodeling, and modifications on histone proteins 3 and 4 [1]. Histone tail modifications include acetylation, phosphorylation, lysine or arginine methylation, ubiquitylation, glycosylation, sumoylation, and ADP-ribosylation [2, 3]. These modifications are individually associated with gene activation or repression and are collectively known as the histone code. Because epigenetic changes are potentially reversible, they provide attractive targets for cancer therapy. Reprogramming of epigenetic controls is also an emerging strategy for in vitro development of stem cells and for generating therapeutically useful differentiated cell types [4]. Demethylating agents currently in use, e.g. azacytidine and decitabine (5-aza-2’-deoxycytidine), are nucleoside analogs. They demethylate promoter DNA slowly because they require incorporation into DNA during cell division and subsequent depletion of DNA methyl transferases (DNMTs) through irreversible binding of these proteins [5]. Their limited efficacy in culture and in treating solid tumors has, however, partially been addressed by co-treatment with histone deacetylase (HDAC) inhibitors such as trichostatin A (TSA) [6].

When exploring compounds for therapeutic functions, the identification of novel properties in lead compounds is an endeavor preceding the subsequent optimization to create a drug. Because the identified lead compounds initially tend to have toxicity/off-target effects and relatively low potency and efficacy, optimization can be a long and expensive process. These two endeavors are discrete. Here, we provide lead compounds so as to begin to explore new properties by which gene desilencing can be accomplished.

From high-throughput cell-based screening, we previously identified eleven compounds that nonspecifically elevated the activity of multiple reporter systems tested [7]. Quinacrine, 1-phenyl-3-(2-thiazolyl)-2-thiourea, piperine, apigenin, and ChemBridge compounds 5100018, 5110235, 5175323, 5175324, 5175328, 5234881, and 5238219 indiscriminately activated gene expression. The activation property was shared among more than one of the following seven reporter systems: Smad4R, RKO p53R, HCT116 p53R, DLD/BFP, CHO-AA8, Shh FF, and Shh REN. Of the eleven agents listed above, four are structurally similar acridine compounds: 5175323, 5175324, 5175328, and quinacrine. These four share a hetero-tri-cyclic functional group known to intercalate into DNA [8] and produced the greatest induction of the reporter systems studied [7]. We therefore set out to determine whether these compounds could be used in cancer cell lines to re-activate methylated and silenced genes that had been implicated in carcinogenesis. We found that acridine compounds could rapidly desilence genes without any apparent requirement for incorporation into DNA. We thus identified a class of lead compounds with novel useful properties which could be optimized in the future for anticancer effects and reprogramming of gene expression.

## RESULTS

### Chemicals nonspecifically enhancing gene expression

To extend our prior results [7], CHO AA8-Luc Tet-Off cells were plated and quadruplicate wells treated with each chemical at each of various concentrations for 18 hours. In these cells, luciferase expression is driven by the constitutively active cytomegalovirus (CMV) promoter. Therefore, luciferase assays were used to measure the effect of treatment on nonspecific gene expression. 5175324 could not be tested because it was not readily available. Five chemicals produced highly robust induction (greater than 10-fold) of the reporter system, indicating indiscriminate elevation of gene expression: TSA, Scriptaid, 5175323, 5175328, and quinacrine. The greatest reporter activity was seen using 0.5 – 5 μg/ml for TSA, 2 – 10 μg/ml for Scriptaid, 2 μg/ml for 5175323, 1 – 2 μg/ml for 5175328 (all replicated multiple times with similar findings), and 5 μg/ml for quinacrine (replicated with similar findings). Lesser induction (less than 10-fold) was observed with other chemicals tested: 5100018, 5234881, and 5238219. These findings were consistent with prior work [7].

**Figure 1:**
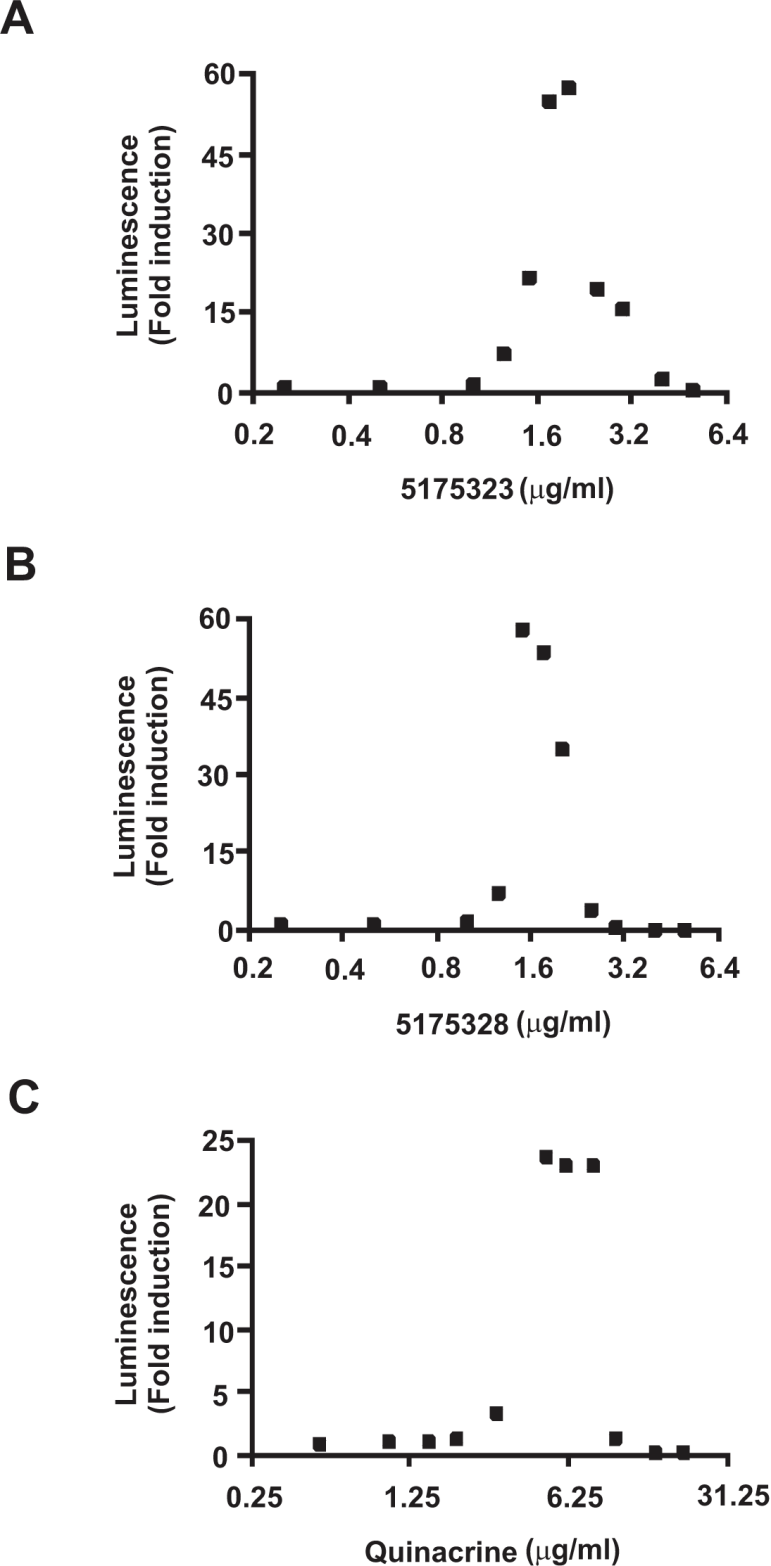
Acridine compounds enhanced nonspecific gene expression Luciferase assays were performed on CHO AA8-Luc Tet-Off cells 18 hours after treatment with compounds 5175323 (A), 5175328 (B), or quinacrine (C). Cells were plated in quadruplicate for each treatment. Data from representative experiments are shown.

Of the five chemicals causing significant induction, TSA and Scriptaid are well established as histone deacetylase inhibitors [9]. The rest belong to the same class of acridine compounds (Table 1). Intrigued by their strong ability to nonspecifically enhance gene expression, we decided to study these compounds (5175323, 5175328, and quinacrine) in greater detail and used dose-response curves to determine the desired range of concentrations for further studies (Fig. 1).

**Table 1:** Compounds

Name/Id	Expanded nomenclature	Structure	Range for reexpression (μg/ml)
5175328	Acridin-9-yl-[4- (4-methylpiperazin- 1-yl)- phenyl]-amine	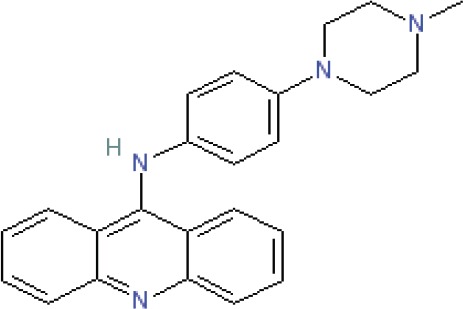	0.5 – 5
5175323	N4- benzo[g]quinolin- 4-yl-N1,N1- diethyl-pentane- 1,4-diamine	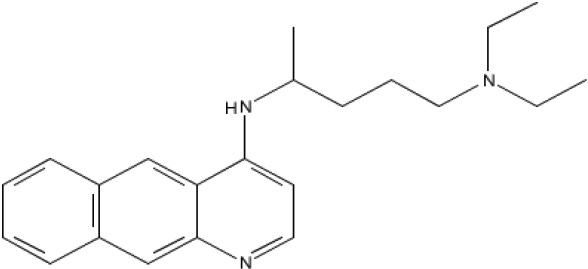	1 – 5
Quinacrine	Quinacrine dihydrochloride dehydrate	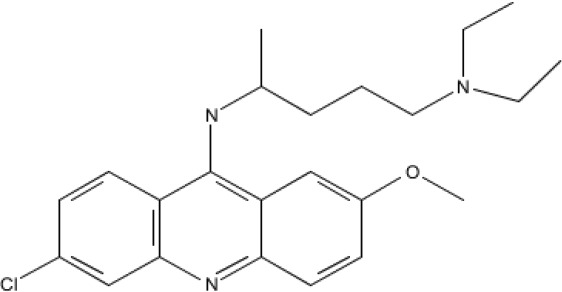	2 - 20

### Attempts to re-activate specific methylated and silenced genes in cancer cell lines

**Figure 2:**
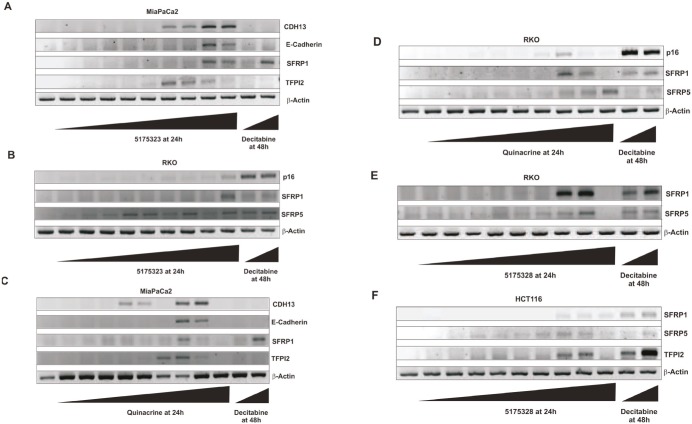
Acridine compounds re-activated methylated and silenced genes in cancer cell lines in a dose-dependent fashion Cells were treated with an acridine compound for 24 hours or with decitabine for 48 hours with drug and medium replaced 24 hours after the beginning of treatment. RNA extracted from the cells was subjected to RT-PCR analysis. β-actin was used as an internal control. A, Compound 5175323 induced gene re-expression in MiaPaCa2 cells. Re-expressed genes: CDH13, E-cadherin, SFRP1, and TFPI2. 5175323 concentrations (μg/ml): 0.01, 0.1, 1, 1.5, 2, 2.5, 3, 4, and 5. Decitabine concentrations (μM): 1 and 5. B, 5175323 induced gene re-expression in RKO cells. Re-expressed genes: p16, SFRP1, and SFRP5. 5175323 concentrations (μg/ml): 0.01, 0.1, 1, 1.5, 2, 2.5, 3, 4, and 5. Decitabine concentrations (μM): 1 and 5. C, Quinacrine induced gene re-expression in MiaPaCa2 cells. Re-expressed genes: CDH13, E-cadherin, SFRP1, and TFPI2. Quinacrine concentrations (μg/ml): 0.01, 0.1, 1, 2, 3, 5, 8, 10, and 20. Decitabine concentrations (μM): 1 and 5. D, Quinacrine induced gene re-expression in RKO cells. Re-expressed genes: p16, SFRP1, and SFRP5. Quinacrine concentrations (μg/ml): 0.01, 0.1, 1, 2, 3, 5, 8, 10, and 20. Decitabine concentrations (μM): 1 and 5. E, 5175328 induced gene re-expression in RKO cells. Re-expressed genes: SFRP1 and SFRP5. 5175328 concentrations (μg/ml): 0.01, 0.05, 0.1, 0.25, 0.5, 1, 1.5, 2, 5. Decitabine concentrations (μM): 1 and 5. F, 5175328 induced gene re-expression in HCT116 cells. Re-expressed genes: SFRP1, SFRP5, and TFPI2. 5175328 concentrations (μg/ml): 0.01, 0.05, 0.1, 0.25, 0.5, 1, 1.5, 2, 5. Decitabine concentrations (μM): 1 and 5.

**Figure 3:**
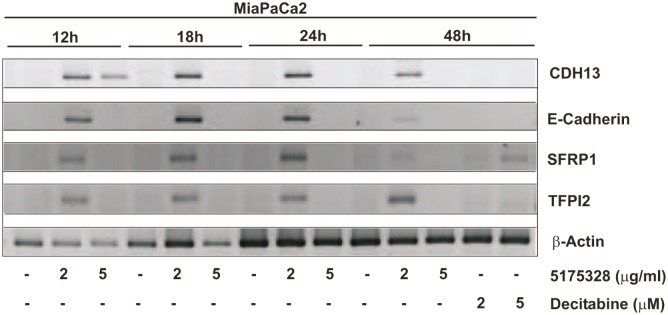
Compound 5175328 re-activated gene expression as early as 12 hours after treatment Re-expressed genes as detected by RT-PCR analysis: CDH13, E-cadherin, SFRP1, and TFPI2.

In an attempt to re-express specific methylation-silenced genes, we treated several randomly chosen cancer cell lines with various concentrations of each acridine compound for 24 hours. We found that 5175323 (Fig. 2A) and quinacrine (Fig. 2C) both re-expressed CDH13, E-cadherin, SFRP1, and TFPI2 in MiaPaCa2 pancreatic cells (replicated with similar findings). 5175323 (Fig. 2B) and quinacrine (Fig. 2D) also re-activated p16, SFRP1, and SFRP5 in RKO colorectal cells (replicated with similar results). 5175328 (Fig. 3) re-expressed CDH13, E-cadherin, SFRP1, and TFPI2 in MiaPaCa2 cells (replicated multiple times with similar findings), but it did not re-activate BNIP3 in the same cells. Similarly, 5175328 (Fig. 2E) desilenced SFRP1 and SFRP5 in RKO cells (replicated with similar observations), but did not re-activate p16 in the same cells. Finally, 5175328 (Fig. 2F) re-expressed SFRP1, SFRP5, and TFPI2 in HCT-116 (colorectal) cells (replicated with similar findings). Sporadic failures to re-express were seen occasionally (Fig. 2C), but re-expression was observed over a continuous range of drug concentration in other experiments. We thus showed that acridine compounds desilenced genes in cancer cells (Table 2).

**Table 2:** Re-expression of methylation-silenced genes

Gene	Silenced in	# of cell lines re-expressing (of # tested)		
		5175328	5175323	Quinacrine
CDH13	MiaPaCa2	1 (1)	1 (1)	1 (1)
E-cadherin	MiaPaCa2	1 (1)	1 (1)	1 (1)
p16	RKO	0 (1)	1 (1)	1 (1)
SFRP1	HCT116, MiaPaCa2, RKO	3 (3)	2 (2)	2 (2)
SFRP5	HCT116, RKO	2 (2)	1 (1)	1 (1)
TFPI2	HCT116, MiaPaCa2	2 (2)	1 (1)	1 (1)

### Specificity: Chemotherapeutic agents do not have a general ability to cause gene re-expression

To address the possibility that gene desilencing was simply a consequence of toxicity or DNA damage, we tested several chemotherapeutic drugs for their ability to induce reporter activity in CHO AA8-Luc Tet-Off cells or desilence genes in MiaPaCa2 cells. Etoposide, 5-fluorouracil (5-FU), mitomycin C, and quercetin caused moderate to negligible induction of the reporter system (less than 10-fold induction) in CHO AA8-Luc Tet-Off cells (Etoposide: 4.5-8.3 fold, 5-fluorouracil (5-FU): 2.4 fold, mitomycin C: 1.5 fold, and quercetin: 1.3 fold). Also, none of these drugs re-activated E-cadherin, SFRP1, or TFPI2 in MiaPaCa2 cells. We concluded that DNA-damaging agents had little ability to enhance gene expression specifically or nonspecifically.

### Rapid onset of effect

Next, we determined the time-course of gene re-activation by 5175328 (our model acridine compound) in MiaPaCa2 cells. We did not see any gene re-expression after 6 hours of treatment. However, 5175328 induced re-expression of CDH13, E-cadherin, SFRP1, and TFPI2 as early as 12 hours after treatment (Fig. 3). The level of re-expression diminished at 48 hours after treatment (Fig. 3).

### Preliminary mechanistic explorations

The focus of these studies was to identify gene-desilencing agents having novel properties, described above. Although outside the fundamental scope of a report of lead compounds, we also performed limited mechanistic explorations that could help explain the class of compounds indentified and orient future research into optimized compounds. It is these later-generation compounds, which would reflect optimization for potency, specificity, and efficacy, that would provide the most suitable agents for defining mechanisms of action. With these caveats, the results of brief mechanistic explorations of the lead compounds are provided below.

### Gene re-expression is accompanied by DNA demethylation and reduced DNMT1 localization at specific promoters

**Figure 4:**
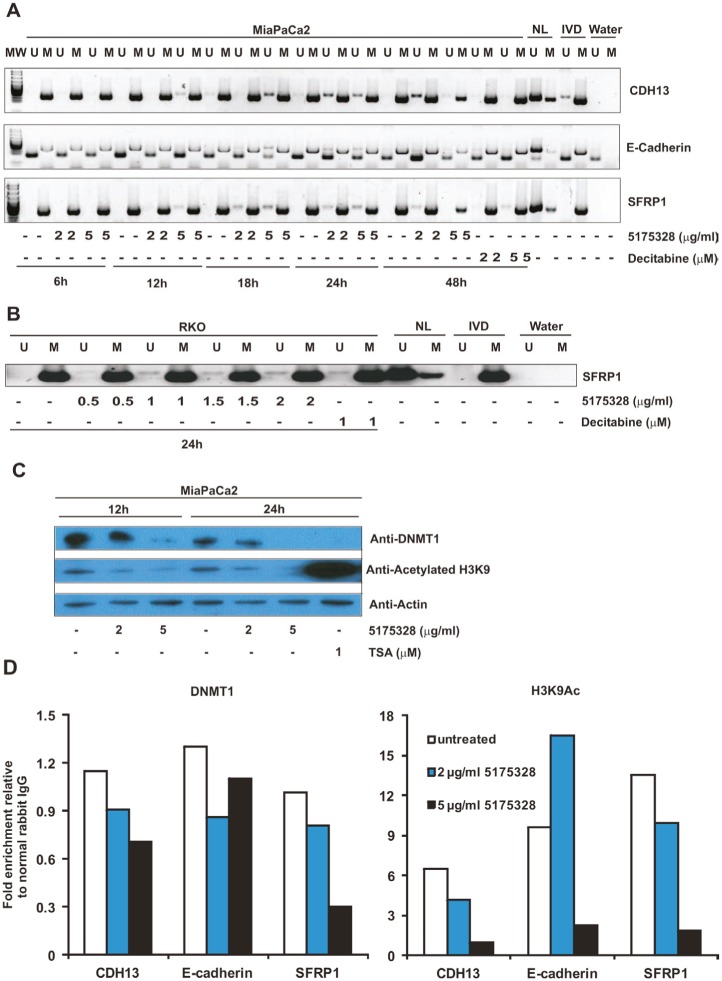
Compound 5175328-mediated gene re-expression appeared to be accompanied by DNA demethylation and DNMT1 depletion at specific promoters A, 5175328 appeared to induce DNA demethylation at CDH13, E-cadherin, and SFRP1 promoters in MiaPaCa2 cells in a dose- and time-dependent manner. U, unmethylated allele; M, methylated allele. Normal human lymphocytes (NL), in vitro methylated DNA (IVD), and water were included as controls. B, 5175328 appeared to induce DNA demethylation at the SFRP1 promoter in RKO cells. U, unmethylated allele; M, methylated allele. Normal human lymphocytes (NL), in vitro methylated DNA (IVD), and water were included as controls. C, DNMT1 quantity and histone acetylation in MiaPaCa2 cells after 5175328 treatment, assayed by western blot. D, DNMT1 localization and histone acetylation at CDH13, E-cadherin, and SFRP1 promoters in MiaPaCa2 cells after 5175328 treatment, assessed by ChIP. PCR assays were performed in triplicate. Data from a representative ChIP experiment are shown.

In an effort to explore possible modes of action, we used methylation-specific PCR (MSP) to examine the methylation status of re-activated promoters after 5175328 treatment since previous studies had established DNA methylation as a mechanism for silencing these genes [10-15]. 5175328 induced dose- and time-dependent demethylation of CDH13, E-cadherin, and SFRP1 promoters in MiaPaCa2 cells. This process began by 6 hours at the CDH13 promoter, 12 hours at the SFRP1 promoter, and 18 hours at the E-cadherin promoter (Fig. 4A). It peaked around 24 hours in all three promoters (Fig. 4A). Evidence for 5175328-dependent demethylation was ambiguous at the desilenced TFPI2 promoter in MiaPaCa2 cells since this gene was only partially methylated prior to treatment. 5175328 consistently induced these changes more rapidly and robustly than decitabine (Fig. 4A). The evidence also suggested dose-dependent demethylation of the SFRP1 promoter in RKO cells after 24 hours of 5175328 treatment (Fig. 4B). However, we could not detect 5175328-mediated demethylation at the re-activated SFRP5 promoter in RKO cells, which was also partially methylated at baseline. Consistent with the failure of 5175328 to re-activate p16 in RKO cells, we did not see evidence for demethylation of the p16 promoter in the same cells. It is conceivable that 5175328 re-activated some genes by alternative mechanisms or that baseline incomplete methylation made detection of small degrees of demethylation challenging.

5175328-induced demethylation of the SFRP1 promoter was confirmed to affect CpG dinucleotides by bisulfite sequencing in RKO and MiaPaCa2 cells (Supplementary Fig. S1). We analyzed 58 CpG sites from the SFRP1 promoter in both cell lines. The average number of demethylated CpG sites per DNA molecule increased after treatment with 5175328. Patches of adjacent demethylated CpG dinucleotides also were observed after treatment. Considering the methylation-specific PCR results and the pattern observed using bisulfite sequencing, 5175328-mediated re-expression of methylation-silenced genes appeared to be accompanied by a degree of rapid promoter demethylation for the majority of genes tested.

We thus wondered whether 5175328 affected global DNMT1 protein quantity, as was observed following treatment of cells with decitabine or azacytidine [16]. We evaluated the effects of 5175328 on DNMT1 protein level and histone acetylation in MiaPaCa2 cells by western blot (replicated with similar observations). TSA-treated MiaPaCa2 cells served as a positive control for histone acetylation (Fig. 4C). We found that the amount of DNMT1 protein decreased in MiaPaCa2 cells after higher-dose 5175328 treatment at 12 hours and 24 hours (Fig. 4C). Interpretation of this finding was, however, complicated by the observation that etoposide and mitomycin C also reduced global DNMT1 protein level in MiaPaCa2 cells without inducing re-expression of methylation-silenced genes. This suggests that depletion of DNMT1 at a global level might not always lead to gene desilencing and may be secondary to inhibition of proliferation, proliferation being associated with DNMT1 expression. We observed a decrease in global histone acetylation after treatment of MiaPaCa2 cells with 5175328 (Fig. 4C), suggesting that inhibition of histone deacetylases was unlikely to be its mechanism of action as this would increase global histone acetylation.

Next, we sought to determine the effect of 5175328 on the localization of DNMT1 to specific re-activated promoters, using the chromatin immunoprecipitation (ChIP) assay (each experiment performed twice with similar findings and PCRs performed in triplicate for each experiment). We saw a decrease in the amount of DNMT1 associated with specific promoters after treatment of MiaPaCa2 cells with 5175328 for 24 hours (Fig. 4D). For example, 5175328 reduced the level of DNMT1 at CDH13, E-cadherin, and SFRP1 promoters (Fig. 4D). A similar pattern of DNMT1 localization was observed at a second site in the SFRP1 promoter. Consistent with the failure to detect any change in demethylation at the TFPI2 promoter, the amount of DNMT1 localized to this promoter did not decrease with 5175328 treatment. Also, in accordance with the pattern of TFPI2 re-expression, the level of histone acetylation peaked after low-dose 5175328 treatment and declined at a higher dose. There was a decrease in the amount of histone acetylation at CDH13 and SFRP1 promoters (Fig. 4D). The pattern of histone acetylation was similar at a second site in the SFRP1 promoter. At the E-cadherin promoter, 5175328 treatment increased the level of histone acetylation at a low dose (correlating with gene re-expression in Fig. 3) and decreased it at a higher dose (Fig. 4D). In summary, 5175328 induced depletion of DNMT1 at specific desilenced promoters, undergoing demethylation. The effect on histone acetylation was variable.

### Acridine compounds intercalate into DNA and inhibit DNMT1 activity in vitro

**Figure 5:**
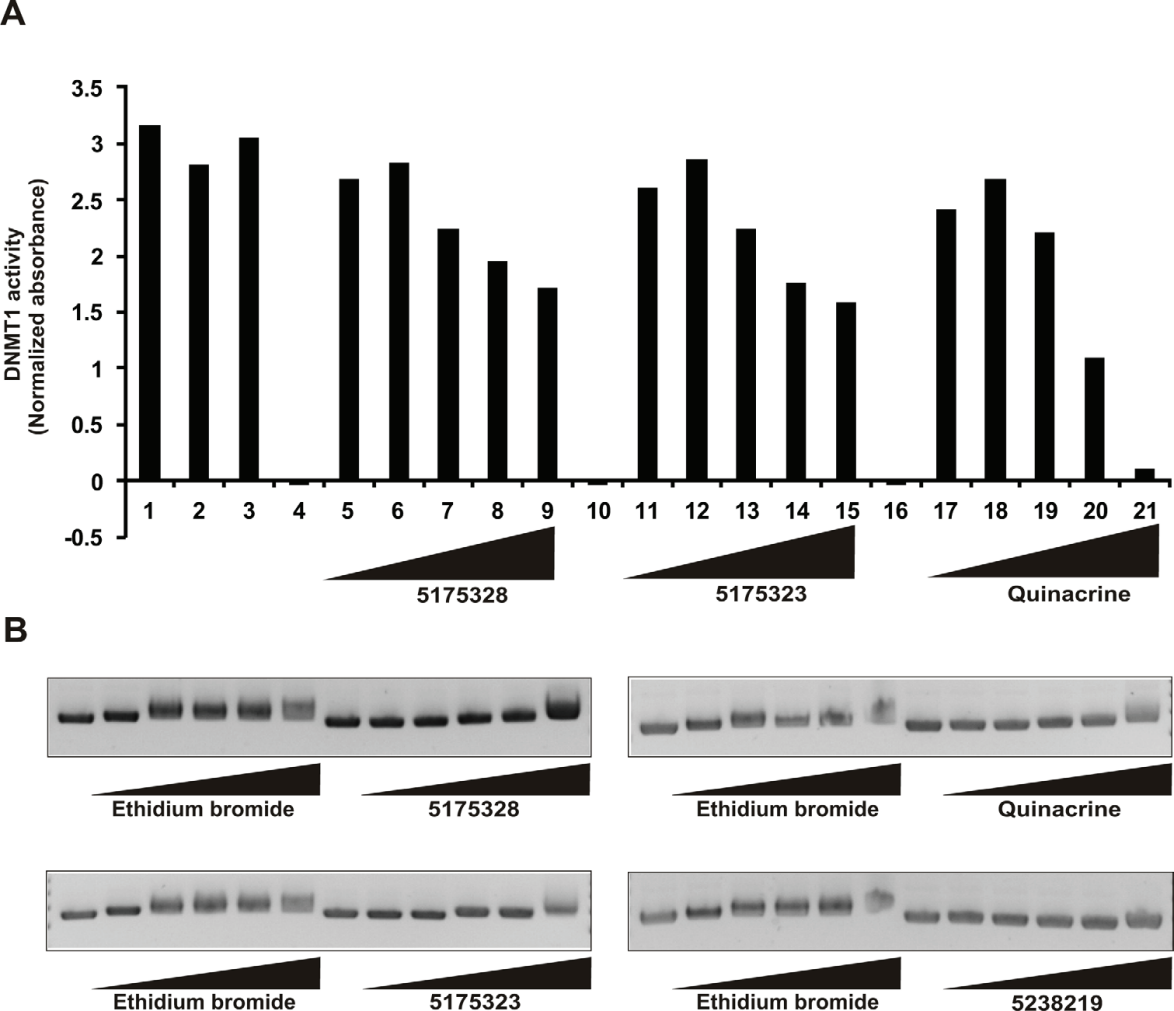
Acridine compounds intercalate into DNA and inhibit DNMT1 activity in vitro A, DNMT1 activity assay in the presence of compounds 5175328, 5175323, and quinacrine. 5-fluorouracil (5-FU) was a negative control. Each assay was performed in duplicate in every experiment. Data from a representative experiment are shown. Normalized absorbance was calculated by subtracting the 655 nm OD from the 450 nm OD. Full description of each bar: DNMT1 (1), DNMT1+DMSO (2), DNMT1 + 100 μM 5FU (3), 10 μg/ml 5175328 without DNMT1 (4), DNMT1+0.1μg/ml 5175328 (5), DNMT1+0.3μg/ml 5175328 (6), DNMT1+1μg/ml 5175328 (7), DNMT1 + 3 μg/ml 5175328 (8), DNMT1 + 10 μg/ml 5175328 (9), 10 μg/ml 5175323 without DNMT1 (10), DNMT1+0.1μg/ml 5175323 (11), DNMT1+0.3μg/ml 5175323 (12), DNMT1+1μg/ml 5175323 (13), DNMT1 + 3 μg/ml 5175323 (14), DNMT1 + 10 μg/ml 5175323 (15), 100 μg/ml quinacrine without DNMT1 (16), DNMT1+1μg/ml quinacrine (17), DNMT1+3μg/ml quinacrine (18), DNMT1+10μg/ml quinacrine (19), DNMT1 + 30 μg/ml quinacrine (20), DNMT1 + 100 μg/ml quinacrine (21). B, DNA intercalation assay of 5175328, 5175323, and quinacrine. Ethidium bromide was a positive control, and 5238219 a negative control. For each compound the following concentrations were used (μg/ml): 0.1, 1, 5, 50, and 500.

Because 5175328 treatment led to the appearance of demethylated promoter DNA, we inquired whether it could directly inhibit DNMT1 in vitro. We assayed the ability of DNMT1 to methylate a DNA substrate in the presence of various concentrations of 5175328, 5175323, and quinacrine (each experiment replicated multiple times with similar findings and assays performed in duplicate for each experiment). Increasing concentrations of 5175328, 5175323, and quinacrine reduced DNMT1 activity (Fig. 5A). 5-fluorouracil, a negative control, had no effect on DNMT1 activity (Fig. 5A). Acridine compounds thus inhibited DNMT1 activity in vitro in a dose-dependent manner.

To explore a potential mechanism by which acridine compounds might inhibit the DNA-interacting enzyme DNMT1, we assayed DNA intercalation in vitro (replicated with similar results). Briefly, closed, circular plasmid DNA was incubated with each compound for 5 minutes. Ethidium bromide was used as a positive control. 5238219 lacking the hetero-tri-cyclic acridine ring, served as a negative control (Fig. 5B). Increasing concentrations of 5175328, 5175323, and quinacrine retarded the migration of plasmid DNA (Fig. 5B). 5175328 at 5 μg/ml caused the same degree of retardation as 5 μg/ml ethidium bromide (Fig. 5B). Higher concentrations of 5175328 resulted in greater retardation, while the effect of ethidium bromide reached a maximum (Fig. 5B). 5175323 at 500 μg/ml and quinacrine at 50 μg/ml induced retardation similar to 5 μg/ml ethidium bromide (Fig. 5B). We concluded that acridine compounds could swiftly intercalate into DNA in a dose-dependent manner.

## DISCUSSION

Our findings suggested that specific acridine compounds rapidly re-activate expression of methylated and silenced genes at μg/ml concentrations (5175328: 0.5 – 5 μg/ml, 5175323: 1 - 5 μg/ml, and quinacrine: 2 – 20 μg/ml). A possible mechanism would be the demethylation of promoter CpG islands due to intercalation of the compound into DNA and subsequent inhibition and depletion of DNMT1 at the desilenced promoter. Other, not yet explored, mechanisms might also be involved, and the process of demethylation may be complex. For example, increasing concentrations of a model acridine compound lead to progressive DNA demethylation and DNMT1 depletion at specific promoters, but gene re-expression was lost beyond an optimal concentration. These higher concentrations were also associated with growth arrest. Since inhibition and depletion of DNMT1 would result in demethylation only if cell division occurred (passive demethylation), progressive demethylation in the absence of cell proliferation would suggest a possible role for non-passive modes of DNA demethylation. Another intriguing observation was the absence of DNMT1 depletion or any detectable change in DNA methylation at the re-activated TFPI2 promoter, allowing for alternative mechanisms of gene desilencing.

Coincidentally, 5175328, a model intercalating agent, is also a highly selective α_2C_-adrenoceptor antagonist at low nM concentrations with anti-depressant and antipsychotic properties [17]. Some of the observed pharmacologic properties of 5175328 at higher doses, however, might include its ability to affect gene expression.

Quinacrine is known to act as a potent inhibitor of histamine N-methyl transferase [18]. It is also reported to have anticancer activity at μM concentrations, synergizing with 5-fluorouracil in inducing apoptosis of colorectal cancer cells [19], rendering hepatocellular carcinoma cells susceptible to TRAIL-induced apoptosis [20], and enhancing toxicity of other chemotherapeutic agents [20]. The mechanisms for these effects are unknown. Our results suggest a new possible mechanism, for quinacrine might exert some of its anticancer effects in part by inhibiting another methyl transferase DNMT1 and affecting DNA methylation, which could alter the expression of genes affecting the sensitivities to other chemotherapies.

Nucleoside DNMT inhibitors such as azacytidine, decitabine (5-aza-2’-deoxycytidine), SG-110 (decitabine prodrug), 5-fluoro-2-deoxycytidine, and zebularine have been used effectively to inhibit DNA methyl transferase and re-express silenced genes. Azacytidine and decitabine have been approved for use in the treatment of myelodysplastic syndrome (MDS), but have properties that may limit their use. They may mutagenize due to DNA strand breaks [5, 21]. They have a short half-life in vivo, and yet require at least 48 hours of treatment in culture even for low-level gene re-activation [6]. Their use is characterized by a slow onset of the demethylated state because they require covalent incorporation into DNA during an initial cell division, prior to any inhibition of DNMTs during a subsequent cell division [5]. Thus, at least three cell divisions are required for demethylation of both strands of DNA to be accomplished. To provide a more efficient reversal of gene silencing, HDAC inhibitors including SAHA (Vorinostat), Belinostat, Entinostat, TSA, Panobinostat, phenyl butyrate, sodium butyrate, valproic acid, Scriptaid, and Romidepsin have been used in conjunction with azacytidine and decitabine [22]. However, HDAC inhibitors cannot be used alone to desilence methylated genes [6]. Polyamine LSD1 (a protein lysine demethylase) inhibitors have also been used to re-express genes, but they were found to be less effective than decitabine [23]. Among histone-lysine-methyl transferase inhibitors, EZH2 inhibitors like 3-Deazaneplanocin A (DZNep) and Sinefungin and G9a (EHMT2) inhibitors like BIX-01294 and 8 (UNC0224) [24] have been used for gene desilencing. Several classes of topoisomerase inhibitors re-activate the dormant allele of Ube3a in neurons [25]. These classes include camptothecin derivatives, indenoisoquinoline derivatives, bis-dioxopiperazine derivatives, the podophyllotoxin derivative etoposide, and the aminoacridine derivative amsacrine [25]. DNA intercalation might plausibly be involved in their desilencing effect, based upon the observations in this manuscript.

Considerable effort has gone into developing putative non-nucleoside DNMT inhibitors as gene desilencing agents. Examples include flavonoids like (-)-epigallocatechin-3-gallate (EGCG) from green tea [26], genistein from soybean [27] [28], quercetin [29], the thiopurine 6-thioguanine [30], disulfiram [21], mitoxantrone [31], psammaplin A [32], hydralazine [33] [34], procainamide [33] [34], procaine [35], N-acetyl-procainamide [36], arsenic trioxide [37], SGI-1027 [38], RG108 [39], and the phosphorothioate antisense oligonucleotide MG98 [40] [41] [42]. Of these, MG98 was abandoned after failure in clinical trials [43] [44]. In subsequent studies, EGCG [45] [46], hydralazine [45], procaine [46], and procainamide [45] were found to have insignificant demethylating activity. RG108 was less effective than azacytidine or decitabine, but comparable to zebularine [46].

A general consensus is that decitabine and azacytidine remain the most potent DNMT inhibitors, superior to other compounds tested [45-47]. Acridine compounds might yet offer some advantages over decitabine and azacytidine as novel non-nucleoside inhibitors of DNMT1 and gene desilencing agents. As a class, they are known to rapidly intercalate into DNA [8]. DNA intercalation by these compounds might mediate DNMT1 inhibition as well as other, not yet identified rapid biochemical events such as active DNA demethylation. Decitabine or azacytidine inhibits DNMT only after covalent incorporation into DNA, this inhibition thus requiring at least two rounds of cell division. Since acridine compounds would not require covalent incorporation into DNA for their activity, demethylation could be observed by methylation-specific PCR after only one round of cell division, as early as 6-12 hours after treatment in vitro. Our study thus identifies a promising class of lead compounds for development of gene-desilencing mehods and reprogramming of gene expression. Among other properties, these compounds are novel, fast-acting, non-nucleoside gene-desilencing agents, and optimized class members might be used to restore expression of epigenetically silenced genes in cancer cells.

## METHODS

### Cell lines and cell culture

MiaPaCa2 (from ATCC), HCT116 (ATCC), RKO (ATCC), and CHO AA8-Luc Tet-Off (Clontech) cells were grown in DMEM medium supplemented with 10% (v/v) fetal bovine serum and 1% (v/v) penicillin/streptomycin. The CHO AA8-Luc Tet-Off cell line was additionally supplemented with 100 μg/ml G418.

### Compounds and drugs

Compounds 5100018, 5175323, 5175328, 5234881, and 5238219 were obtained from ChemBridge. 5-Aza-2’-deoxycytidine (decitabine), etoposide, 5-fluorouracil (5-FU), mitomycin C, quercetin, quinacrine, Scriptaid [7, 9], and TSA were from Sigma. Ethidium bromide was from Fisher Biotech. Compounds 5175323, 5175328, and quinacrine are further described in Table 1.

### Luciferase reporter assay

Luciferase reporter assay was performed with the Promega Firefly Luciferase Assay System according to the manufacturer’s protocol. Briefly, cells were plated in quadruplicate, incubated with each compound for 18 hours, and then lysed for luciferase assay. We used the PerkinElmer Microbeta Trilux plate reader to measure luminescence.

### Reverse transcriptase polymerase chain reaction (RT-PCR)

Total RNA was extracted from cell populations using the Qiagen RNeasy Mini Kit and subjected to DNAse I (Invitrogen) digestion to remove contaminating DNA. 1 μg of RNA was reverse-transcribed in a 20 μl volume (SuperScript III First-Strand Synthesis System, Invitrogen). In a parallel tube, 1 μg of RNA was treated with the cDNA synthesis reagents without the reverse transcriptase (RT) enzyme in a 20 μl volume (the “no RT control”). 1 μl of the cDNA mixture or “no RT control” was subjected to PCR amplification for 35 cycles to evaluate expression of genes of interest. Primer sequences used in RT-PCR reactions and corresponding annealing temperatures are listed in Supplementary Table S1.

### Methylation-specific PCR (MSP)

Genomic DNA was extracted from cell populations (Qiagen QIAmp DNA Mini Kit). EZ DNA Methylation Kit (Zymo Research) was used for bisulfite treatment of DNA. To determine the methylation status of promoter regions, each sample of bisulfite-modified DNA was subjected to 35 cycles of PCR amplification with an annealing temp of 60° C using one primer pair specific for methylated DNA and another pair for unmethylated DNA. Primers used for MSP reactions are provided in Supplementary Table S2.

### Bisulfite sequencing

Bisulfite-treated DNA (~100 ng) was amplified with primers encompassing the SFRP1 transcriptional start site: SFRP1-BSeq-forward, 5′-TGGTTTTGTTTTTTAAGGGGTGTTGAGT -3′; CTNNA1-BSeq-reverse, 5′-TCCTACCRCAAACTTCCAAAAACCT-3’ with an annealing temp of 60° C. This amplified a 429 bp region of the CpG island, containing 59 CpG sites. PCR products were separated from reagents using PCR cleanup and cloned into the pCR2.1-TOPO vector (Invitrogen). Plasmids from single colonies were purified using QIAprep Spin Miniprep Kit (Qiagen) and were sequenced with M13 reverse primers. All CpG sites (58) from the amplicon (excluding primer sequences) were analyzed.

### Western blot

To prepare whole-cell extracts, cells were lysed by rocking in a detergent (50 mM Tris-HCl, 150 mM NaCl, 1mM EDTA, 1% Triton X-100, and a protease inhibitor cocktail (Roche)) for an hour at 4°C and clarified by centrifugation. The supernatant was recovered as a whole-cell extract. To prepare nuclear extracts, cells were lysed by gentle hypotonic treatment (10 mM HEPES pH 7.9, 1.5 mM MgCl_2_, 10 mM KCl, 0.5% (v/v) NP-40, and protease inhibitor) and gently centrifuged. The supernatant was removed as the cytoplasmic extract. The nuclear pellet was resuspended (50 mM Tris-HCl, 150 mM NaCl, 1mM EDTA, 1% Triton X-100, and protease inhibitor). The protein concentration was determined by the DC protein assay (Bio-Rad). After adding a denaturant (2% (m/v) sodium dodecyl sulfate (SDS), 10% (v/v) glycerol, 0.002% (m/v) bromophenol blue, 2 mM EDTA, 50 mM Tris pH 6.8, and 1% (v/v) β-mercaptoethanol), samples were boiled and resolved on a NuPAGE 4-12% Bis-Tris gel (Invitrogen). Proteins were transferred to a polyvinylidene fluoride (PVDF) membrane (Pierce). Blots were incubated with primary antibodies: anti-DNMT1 (Sigma), anti-actin (Santa Cruz), or anti-acetyl histone3 lysine9 (Millipore), followed by horseradish peroxidase (HRP)-conjugated secondary antibodies (Santa Cruz): anti-rabbit IgG, anti-goat IgG, or anti-mouse IgG. Membranes were developed with the Immobilon substrate (Millipore). Chemi-luminescence signals were captured on HyBlot CL film (Denville).

### Chromatin immunoprecipitation (ChIP) assay

Cells were crosslinked in 1% formaldehyde for 10 minutes, followed by addition of glycine for 5 minutes to quench unreacted formaldehyde. Cells were subsequently processed with the EZ-ChIP Chromatin Immunoprecipitation Kit (Millipore) according to the manufacturer’s instructions. Cross-linked protein-DNA complexes were captured with anti-DNMT1 (Sigma), anti-acetyl histone3 lysine9 (Millipore), or normal rabbit IgG (Millipore) antibodies. Real-time quantitative PCR (Bio-Rad iCycler) was performed to determine the relative abundance of the promoter DNA sequence associated with the protein of interest. Primers used for ChIP real-time quantitative PCRs and corresponding annealing temperatures are provided in Supplementary Table S3.

### Assay for DNMT activity

Recombinant DNMT1 was obtained from Active Motif. The DNMT activity assay was performed using the EpiQuik DNMT Activity Assay Ultra Kit (Epigentek) according to the manufacturer’s instructions.

### Assay for DNA intercalation

Closed, circular pRLSV40 plasmid (3705 bp) (0.5 μg) was incubated with various concentrations of each chemical in 10 mM Tris-HCl, pH 7.5 at 37° C for 5 minutes [48]. An aliquot of the reaction mixture was analyzed by electrophoresis on a 0.6% agarose gel at 4° C. The gel was incubated in ethidium bromide for 30 minutes and photographed under UV light.

## Supplementary Figures and Tables




