# Supplementary material for: DNA-intercalators Causing Rapid Re-expression of Methylated and Silenced Genes in Cancer Cells
Source: Oncotarget. 2013 Feb 26;4(2):298–309. doi: 10.18632/oncotarget.863 (PMC3712575; doi:10.18632/oncotarget.863)
Supplement: Supplementary file 1 [file oncotarget-04-298-s001.pdf]

DNA-intercalators Causing Rapid Re-expression of Methylated and Silenced Genes in Cancer Cells - Hossain et al

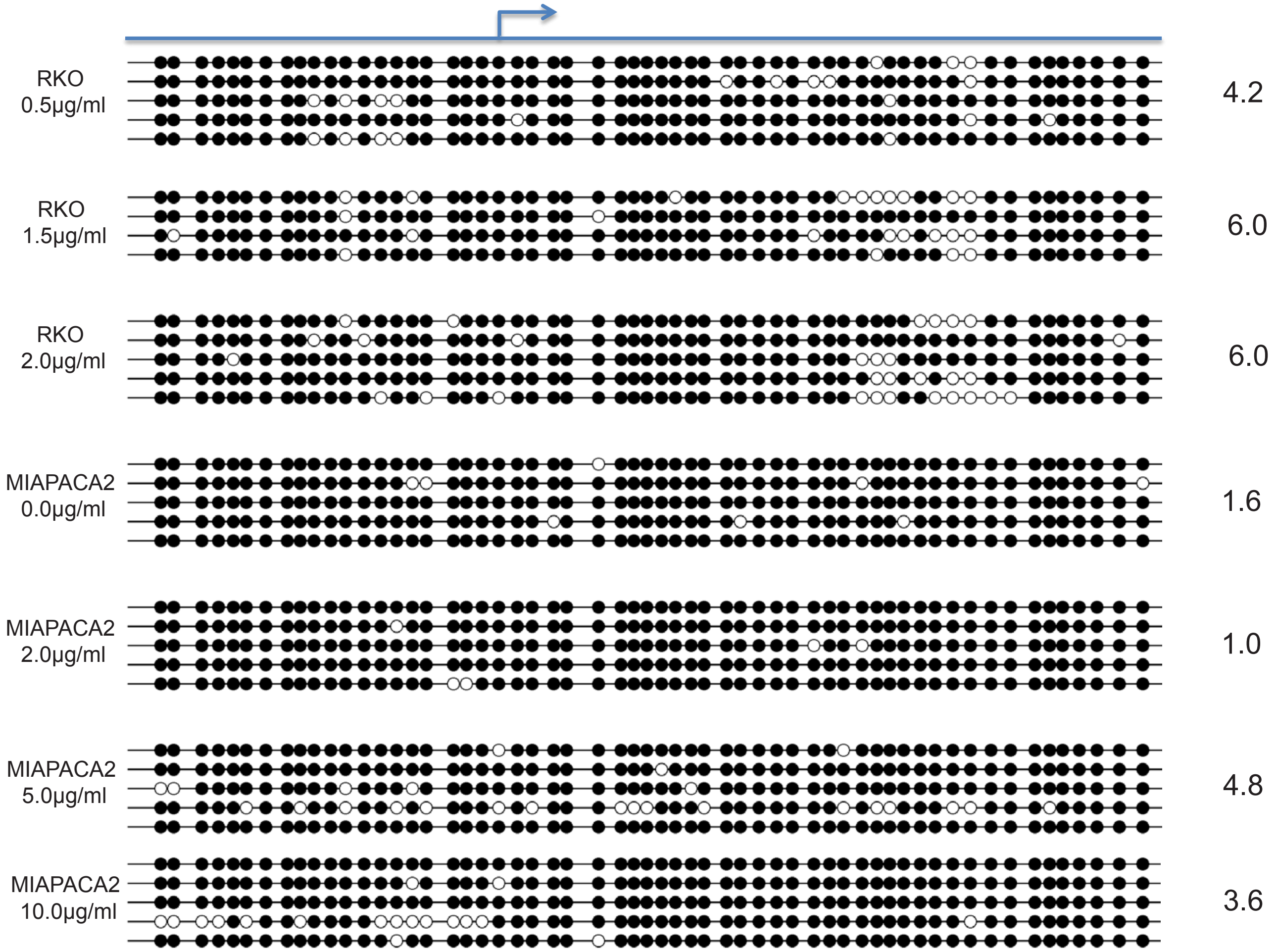

Figure S1: Compound 5175328 appeared to demethylate the SFRP1 promoter in a dose-dependent manner. RKO or MiaPaCa2 cells were treated with 5175328 for 24 hours. Bisulfite-treated genomic DNA from these cells was PCR-amplified with primers designed to amplify the CpG-rich region of the SRRP1 promoter, containing 59 CpG sites. PCR products were cloned into the pCR2.1-TOPO vector, and plasmids from single colonies were sequenced. All CpG sites (58) from the amplicon (excluding primer sequences) were analyzed. Each line represents the DNA sequence of a single clone. The arrow at the top indicates the transcriptional start site. Each filled circle represents a methylated CpG site, and an empty circle indicates an unmethylated CpG site, arrayed in their natural order. The numbers on the right indicate the average number of demethylated CpG sites per DNA molecule. Increased numbers of unmethylated sites and clusters of such sites (empty circles) were seen after 5175328 treatment, indicating sites and patches of demethylated DNA respectively.
