# Supplementary material for: DNA-intercalators Causing Rapid Re-expression of Methylated and Silenced Genes in Cancer Cells
Source: Oncotarget. 2013 Feb 26;4(2):298–309. doi: 10.18632/oncotarget.863 (PMC3712575; doi:10.18632/oncotarget.863)
Supplement: Supplementary file 2 [file oncotarget-04-298-s002.doc]

DNA-intercalators Causing Rapid Re-expression of Methylated and Silenced Genes in Cancer Cells - Hossain et al

Table S1: List of Primers for RT-PCR

| **Name** | **Sequence (5’-3’)** | **Product Size (Annealing Temperature ⁰C)** |
| --- | --- | --- |
| β-actin forward | AGAAAATCTGGCACCACACC | 435 bp (56) |
| β-actin reverse | CCATCTCTTGCTCGAAGTCC |  |
| CDH13 forward | TTCAGCAGAAAGTGTTCCATAT | 204 bp (57) |
| CDH13 reverse | GTGCATGGACGAACAGAGT |  |
| E-cadherin forward | CCTGCCACCCTGGCTTTGAC | 369 bp (68) |
| E-cadherin reverse | TGAGGCCAGGAGAGGAGTTG |  |
| p16 forward | CGGAAGGTCCCTCAGACATC | 385 bp (60) |
| p16 reverse | TCATGAAGTCGACAGCTTCCG |  |
| SFRP1 forward | CTGGCCCGAGATGCTTAAG | 189 bp (60) |
| SFRP1 reverse | TATTTTCATCCTCAGTGCAAAC |  |
| SFRP5 forward | TGGAGCCCAGAAAAAGAAGA | 247 bp (56) |
| SFRP5 reverse | GCAGGGGTAGGAGAACATGA |  |
| TFPI2 forward | CCAGATGAAGCTACTTGTATG | 209 bp (63) |
| TFPI2 reverse | GCACATGCACGTTTGCAATC |  |

**Table S2: List of Primers for MSP**

| **Name** | **Sequence (5’-3’)** | **Product Size** |
| --- | --- | --- |
| CDH13 MSP U F | GGTTTTTATGGAAAATATGTTTAGTGTAGTTGT | 115 bp |
| CDH13 MSP U R | CAAAAAAACACACAAAACAAACAAAATTCTCA |  |
| CDH13 MSP M F | CGGAAAATATGTTTAGTGTAGTCGC | 98 bp |
| CDH13 MSP M R | GCACAAAACGAACGAAATTCTCG |  |
| E-cadherin MSP U F | TGGTTGTAGTTATGTATTTATTTTTAGTGGTGTT | 120 bp |
| E-cadherin MSP U R | ACACCAAATACAATCAAATCAAACCAAA |  |
| E-cadherin MSP M F | TGTAGTTACGTATTTATTTTTAGTGGCGTC | 112 bp |
| E-cadherin MSP M R | CGAATACGATCGAATCGAACCG |  |
| p16 MSP U F | TTATTAGAGGGTGGGGTGGATTGT | 151 bp |
| p16 MSP U R | CAACCCCAAACCACAACCATAA |  |
| p16 MSP M F | TTATTAGAGGGTGGGGCGGATCGC | 150 bp |
| p16 MSP M R | GACCCCGAACCGCGACCGTAA |  |
| SFRP1 MSP U F | GTTTTGTAGTTTTTGGAGTTAGTGTTGTGT | 135 bp |
| SFRP1 MSP U R | CTCAACCTACAATCAAAAACAACACAAACA |  |
| SFRP1 MSP M F | TGTAGTTTTCGGAGTTAGTGTCGCGC | 126 bp |
| SFRP1 MSP M R | CCTACGATCGAAAACGACGCGAACG |  |
| SFRP5 MSP U F | GTAAGATTTGGTGTTGGGTGGGATGTTT | 141 bp |
| SFRP5 MSP U R | AAAACTCCAACCCAAACCTCACCATACA |  |
| SFRP5 MSP M F | AAGATTTGGCGTTGGGCGGGACGTTC | 136 bp |
| SFRP5 MSP M R | ACTCCAACCCGAACCTCGCCGTACG |  |
| TFPI2 MSP U F | CCCACATAAAACAAACACCCAAACCA | 93 |
| TFPI2 MSP U R | TGGTTTGTTGGGTAAGGTGTTTG |  |
| TFPI2 MSP M F | GTTCGTTGGGTAAGGCGTTC | 86 |
| TFPI2 MSP M R | CATAAAACGAACACCCGAACCG |  |

**Table S3: List of Pri**mers for ChIP

| **Name** | **Sequence (5’-3’)** | **Product Size (Annealing Temperature ⁰C)** |
| --- | --- | --- |
| CDH13 ChIP forward | CGATCCAGATCCCAGCTTTG | 165 bp (60) |
| CDH13 ChIP reverse | GTGCTTCCCTGCCATCTCAC |  |
| E-cadherin ChIP forward | TCGAACCCAGTGGAATCAGAAC | 100 bp (60) |
| E-cadherin ChIP reverse | CCACCCGGCCTCGCATAGAC |  |
| SFRP1 ChIP forward 1 | AATTTCATGGGTTTGCAAGTATGA | 158 bp (52) |
| SFRP1 ChIP reverse 1 | TAAATAAAAGGGGGAGGAGGAAAG |  |
| SFRP1 ChIP forward 2 | ACCAACAGCCCTTTAGACTAG | 100 bp (60) |
| SFRP1 ChIP reverse 2 | CGCTTCAGCCTTCCTGAAC |  |
| TFPI2 ChIP forward | CGCCCTGAGGGAGCGTTTG | 195 bp (60) |
| TFPI2 ChIP reverse | TGAAGGTCCATTGCAACGAATC |  |
